# Chemical-induced disease relation extraction with various linguistic features

**DOI:** 10.1093/database/baw042

**Published:** 2016-04-06

**Authors:** Jinghang Gu, Longhua Qian, Guodong Zhou

**Affiliations:** ^1^Natural Language Processing Lab, School of Computer Science and Technology, Soochow University, 1 Shizi Street, Suzhou, China, 215006

## Abstract

Understanding the relations between chemicals and diseases is crucial in various biomedical tasks such as new drug discoveries and new therapy developments. While manually mining these relations from the biomedical literature is costly and time-consuming, such a procedure is often difficult to keep up-to-date. To address these issues, the BioCreative-V community proposed a challenging task of automatic extraction of chemical-induced disease (CID) relations in order to benefit biocuration. This article describes our work on the CID relation extraction task on the BioCreative-V tasks. We built a machine learning based system that utilized simple yet effective linguistic features to extract relations with maximum entropy models. In addition to leveraging various features, the hypernym relations between entity concepts derived from the Medical Subject Headings (MeSH)-controlled vocabulary were also employed during both training and testing stages to obtain more accurate classification models and better extraction performance, respectively. We demoted relation extraction between entities in documents to relation extraction between entity mentions. In our system, pairs of chemical and disease mentions at both intra- and inter-sentence levels were first constructed as relation instances for training and testing, then two classification models at both levels were trained from the training examples and applied to the testing examples. Finally, we merged the classification results from mention level to document level to acquire final relations between chemicals and diseases. Our system achieved promising *F*-scores of 60.4% on the development dataset and 58.3% on the test dataset using gold-standard entity annotations, respectively.

**Database URL**: https://github.com/JHnlp/BC5CIDTask

## Introduction

With the rapid accumulation of the scientific literature, there is an increasing interest in extracting semantic relations between chemicals and diseases described in text repositories, as they play an important role in many areas in healthcare and biomedical research (1–3).

Identification of chemical–disease relations (CDRs), such as mechanistic and biomarker/correlative relations from the literature, can be helpful in developing chemicals for therapeutics and improving studies on chemical safety and toxicity. Nevertheless, manual annotation of CDR from unstructured free text into structured knowledge, such as the Comparative Toxicogenomics Database (CTD) project (4), is costly and inefficient to keep up with the rapid growth of the biomedical literature.

Although some previous attempts (5–8) have been made on automatic biomedical information extraction from free-text corpora, many tasks such as identifying relevant biomedical concepts (9–11) and extracting relations between biomedical entities (12, 13) still remain challenging. In addition, few relation extraction tools in biomedical domain are freely available, and their application to real-world scenarios is limited.

In order to promote research on these issues, the BioCreative-V community proposed a challenging task of automatic extraction of CDR from the biomedical literature. The task was aimed to provide practical benefits to biocuration, and consisted of two specific subtasks:

- Disease Named Entity Recognition and Normalization (DNER). The primary step for automatic CDR extraction is DNER, which was found to be difficult in previous BioCreative tasks (14, 15). For this subtask, participants were given the abstracts of raw PubMed articles and asked to return normalized concept identifiers for disease entities. In this subtask, both chemicals and diseases were described using the Medical Subject Headings (MeSH)-controlled vocabulary.
- Chemical-induced disease (CID) relation extraction. Participants were provided with the same raw text as DNER, and asked to return a ranked list of chemical and disease entity pairs with normalized concept identifiers with which CIDs were associated in the abstract. For this task, the CID relations referred to two specific types of interactions between a chemical and a disease, i.e. molecular mechanism relation and biomarker relation.

In the CID subtask, the molecular mechanistic relationship between a chemical and a disease means that the chemical may play a role in the etiology of the disease (e.g. exposure to chemical X causes lung cancer). The biomarker relationship between a chemical and a disease indicates that the chemical correlates with the disease (e.g. increased abundance in the brain of chemical X correlates with Alzheimer disease).

In particular, the CID relation is determined between two entities, i.e. a chemical and a disease, rather than two mentions, which means that the relationship can be derived either from one sentence, or from a discourse spanning several sentences. Since chemical and disease entities may have multiple mentions scattered in different sentences in a document, the CID relations are essentially interpreted at document level. We regard the case as ‘Intra-sentence Level’ where two mentions of chemical and disease entities occur in the same sentence or as ‘Inter-sentence Level’ otherwise. The CID relation extraction task can be boiled down from document level to mention level, taking the following sentences into consideration:

- *After 2 individuals with* ***psoriasis*** *developed a* ***capillary leak syndrome*** *following treatment with oral* ***sirolimus*** *lesional skin cells and activated peripheral blood cells were analyzed for induction of apoptosis.*
- *OBSERVATIONS: A keratome skin specimen from 1 patient with* ***sirolimus****-induced* ***capillary leak syndrome*** *had a 2.3-fold increase in percentage of apoptotic cells (to 48%) compared with an unaffected* ***sirolimus****-treated patient with* ***psoriasis*** *(21%).*
- *Because patients with severe* ***psoriasis*** *may develop* ***capillary leak*** *from various systemic therapies, clinical monitoring is advisable for patients with* ***inflammatory*** *diseases who are treated with immune modulators.*

Above sentences are extracted from the same document (PMID: 10328196). Among them, the texts in bold are mentions of chemical or disease entities, where ‘***sirolimus***’ refers to a chemical entity whose concept identifier is D020123 (C1), ‘***capillary leak syndrome***’ and ‘***capillary leak***’ represent the same disease entity whose concept identifier is D019559 (D1), ‘***psoriasis***’ refers to a disease entity whose concept identifier is D011565 (D2) and ‘***inflammatory***’ refers to another disease entity with the concept identifier of D007249 (D3). The chemical C1 has intra-sentence level co-occurrence with diseases D1 and D2, respectively, in both sentences (*a*) and (*b*), while it has inter-sentence level co-occurrence with the disease D3. Between C1 and D1, there is a true CID relation.

The BioCreative-V task required all participants to use web services for online evaluation. One of the benefits of online evaluation was that the task organizer could remotely request text-mined results in real-time without additional investment in text-mining tool adoption and technical infrastructure.

In this article, we report our approach to the CID relation extraction subtask of the BioCreative-V CDR task. Our primary goal was to develop a relation extraction system that could scale well over free text documents using machine learning approaches. We first extracted CID relations at mention level by using two maximum entropy based classifiers with various features for intra- and inter-sentence levels, respectively. Then we merged these results to obtain CID relations between entities at document level. Additionally, the hypernym relationship between entity concepts was leveraged during the training stage to remove negative instances as well as during the testing stage to filter positive instances in order to further improve the extraction performance. Currently, we mainly focused on the CID relation extraction at intra-sentence level using general natural language processing technologies.

## Methods

**Figure 1.**
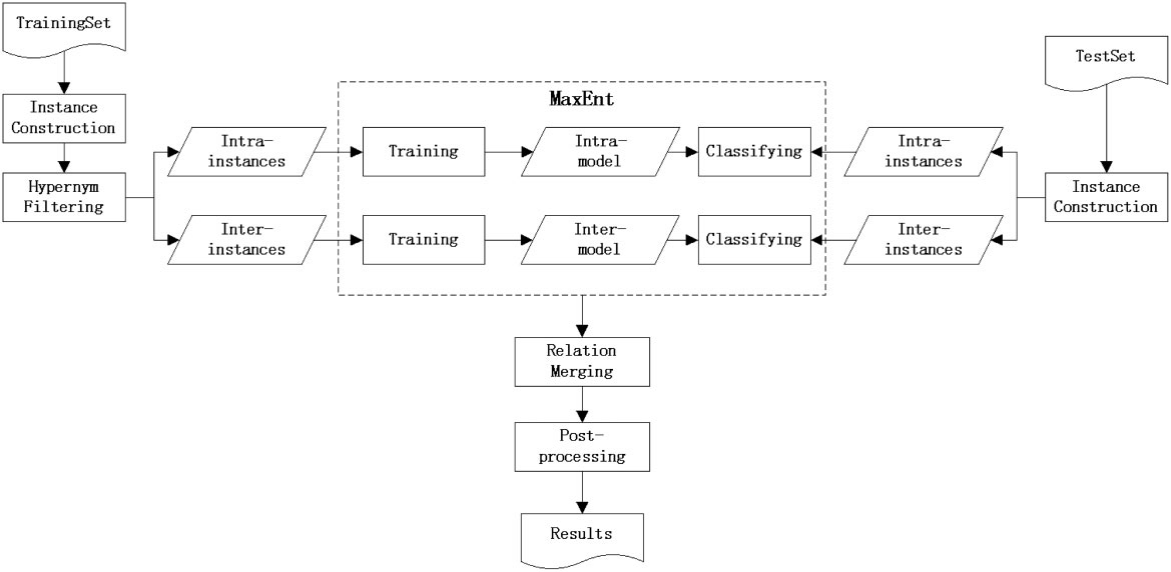
System workflow diagram.

This section illustrates our supervised learning approach to the CID relation extraction. Figure 1 presents the overall architecture of our system. The system took raw text documents in the PubTator format (16, 17) as input, then extracted CID relations at mention level by maximum entropy classifiers and finally merged the classification results to acquire relations between entities at document level. The whole process of our approach can be divided into five sequential steps as follows:

### Relation instance construction

In this step, pairs of chemical and disease mentions in the form of* <chemical mention, disease mention>* were extracted as relation instances by employing several heuristic filtering rules on both training and testing datasets.

All the instances were generated from chemical and disease mentions in the same document in a pairwise way, i.e. if a document contained *m* different chemical mentions and *n* different disease mentions, there were *m *×* n* different chemical and disease mention pairs. These mention pairs were pooled into two groups at intra-sentence level and inter-sentence level, respectively. The former means that a mention pair was from the same sentence, while the latter means otherwise. After applying different heuristic filtering rules, the left mention pairs were taken as relation instances. In addition, since the CID task concerned fine-grained relationships between chemicals and diseases, the general relations that were not disease specific should be excluded (e.g. toxicity) (18). We removed relation instances that were not disease specific by following the annotation guideline of CDR corpus (18) according to the MeSH-controlled vocabulary [e.g. ‘Drug-Related Side Effects and Adverse Reactions’ (D064420)].

When constructing training instances, it was assumed that if two entities were involved in the same relation, any sentence that contained these two entity mentions would express that relationship. That is, if the relation between the two entities of the mention pair was annotated as true, we would take this mention pair as a positive instance; otherwise, it would be taken as a negative instance. The filtering rules for intra-sentence level and inter-sentence level instances are detailed in Section ‘Relation instance construction for intra-sentence level’ and Section ‘Relation instance construction for inter-sentence level’, respectively.

#### Relation instance construction for intra-sentence level

#### 

Prior to relation extraction, relation instances at intra-sentence level for both training and testing processes should be constructed. For this purpose, we applied some simple yet effective heuristic rules as follows:

- The token distance between the two mentions in an instance should be less than *k* (here we set *k* to 10 empirically).
- If there are multiple mentions in a sentence that refer to the same entity, the nearest pair of chemical and disease mentions should be kept as the instance.
- Any mention that occurs in parentheses should be ignored.

For instance, there are four mentions in sentence (*b*): two refer to the same chemical entity C1, one refers to the disease entity D1, and one refers to another disease entity D2. The first mention of C1 and the mention of D1 will constitute an intra-sentence level instance of <***sirolimus***, ***capillary leak syndrome***>, whose token distance is 3. The second mention of C1 and the mention of D2 will constitute another instance of <***sirolimus*, *psoriasis***>, whose token distance is 5. Other combinations of chemical and disease mentions will be discarded because of the overlong token distance.

#### Relation instance construction for inter-sentence level

The relation instance construction at inter-sentence level for training and testing complied with the following heuristic rules:

- Only the entities that are not involved in any intra-sentence level instance are considered at inter-sentence level.
- The sentence distance between two mentions in an instance should be less than *n* (here we set *n* to 3 empirically).
- If there are multiple mentions that refer to the same entity, keep the pair of chemical and disease mentions that results in the nearest distance.

For instance, there are three mentions in sentence (*c*), referring to three different disease entities, and there is no chemical mention in the sentence. The mention of ‘***inflammatory***’ will be paired with the mention of ‘***sirolimus***’ to construct an instance at inter-sentence level, since only inter-sentence level co-occurrence is found between these two entities. However, the mentions of ‘***psoriasis***’ and ‘***capillary leak***’ will be omitted since the pairs of the two entities to which these mentions refer have intra-sentence co-occurrence with chemicals in other sentences.

### Hypernym filtering for training instances

In some cases, there was a hypernym/hyponym relationship between concepts of diseases or chemicals, where a concept was subordinate to another more general concept. However, the goal of the CID subtask aimed to automatically extract the relationships between the most specific diseases and chemicals, i.e. the relations between hyponym concepts should be considered rather than hypernym concepts. For instance, taking the following sentences into consideration:

d. ***Carbamazepine****-induced* ***cardiac dysfunction****.*
e. *A patient with sinus* ***bradycardia*** *and* ***atrioventricular block****, induced by* ***carbamazepine****, prompted an extensive literature review of all previously reported cases.*

Above two sentences are extracted from the same document (PMID: 1728915), where (*d*) is from the title and (*e*) is from the abstract. In the sentences, the texts in bold are mentions of chemical or disease entities, where ‘***Carbamazepine***’ and ‘***carbamazepine***’ both stand for a chemical entity whose concept identifier is D002220 (C2); ‘***cardiac dysfunction***’ stands for a disease whose concept identifier is D006331 (D4); ‘***bradycardia***’ stands for a disease whose concept identifier is D007249 (D5); and ‘***atrioventricular block***’ stands for a disease with the concept identifier of D054537 (D6). In the sentences, there are three CID relations, i.e. C2-D4, C2-D5 and C2-D6. The latter two are more specific than the first one because D4 is the hypernym of both D5 and D6. According to the annotation guideline of the corpus, the relations of C2-D5 and C2-D6 should be annotated as true while the relation of C2-D4 should not.

However, from the perspective of relation extraction, the phrase ‘***Chemical****-induced* ***Disease***’ in sentence (*d*) is a typical pattern for positive relation instance. If this instance is regarded as a negative instance in the training data, it will definitely confuse the classifier and cause its performance deterioration. Therefore, in the hypernym filtering step, we used the MeSH tree numbers of concepts to determine the hypernym relationship between entities in a document and removed those negative instances that involved entities which were more general than other entities already participating in the positive ones. For example, the C2-D4 instance in sentence (*d*) would be removed from the negative training data. However, we did not take a more aggressive step to include it as a positive one.

### Relation extraction

CID relation extraction could be recast as a binary classification problem. The training instances were fed into a learner to derive a classification model which was in turn used to predict the relationship for the test instances. Typically, both the training and test instances were represented as feature vectors. Therefore, the feature selection played an important role in feature-based supervised learning. In the following sections, we propose various lexical features and dependency features for relation recognition at both intra- and inter-sentence levels.

#### Feature extraction for intra-sentence level

Various lexical, syntactic and semantic features had been leveraged in (19) to extract semantic relations between entities from newswire texts. Following the same line, both lexical and dependency features could also be used in biomedical domain, among which the lexical features we used were similar to those in (19).

♦ Lexical features (LEX):
  - CMTXT: chemical mention text
  - CBOW: bag-of-words of chemical mention
  - CPOS: part of speech of chemical mention
  - DMTXT: disease mention text
  - DBOW: bag-of-words of disease mention
  - DPOS: part of speech of disease mention
  - WVNULL: when no verb in between
  - WVFL: when only one verb in between
  - WVTFL: the verb when only one verb in between
  - WVLFL: verb lemma when only one verb in between
  - WVF: first verb in between when at least two verbs in between
  - WVFLM: first verb lemma in between when at least two verbs in between
  - WVL: last verb in between when at least two verbs in between
  - WVLLM: last verb lemma in between when at least two verbs in between
  - WVO: other verbs in between except the first and last verbs
  - WVOLM: other verb lemmas in between except the first and last verbs
  - WBF: first word in between when at least two words in between
  - WBFLM: first word lemma in between when at least two words in between
  - WBFPOS: part of speech of the first word in between when at least two words in between
  - WBL: last word in between when at least two words in between
  - WBLLM: last word lemma in between when at least two words in between
  - WBLPOS: part of speech of the last word in between when at least two words in between
  - WBNULL: when no word in between
  - WBFL: when only one word in between
  - WBTFL: the word when only one word in between
  - WBLFL: word lemma when only one word in between
  - WBTPOS: part of speech of the word in between when only one word in between
  - BM1F: first word before the first mention
  - BM1FL: first word lemma before the first mention
  - BM1L: second word before the first mention
  - BM1LL: second word lemma before the first mention
  - AM2F: first word after the second mention
  - AM2FL: first word lemma after the second mention
  - AM2L: second word after the second mention
  - AM2LL: second word lemma after the second mention
  - WINT: whether the instance occurs in title
♦ Dependency features (DEP):
  - DPR2C: dependency path from root to chemical mention
  - DPR2D: dependency path from root to disease mention
  - DPC2D: dependency path from chemical mention to disease mention
  - DPC2DR: dependency path from chemical mention to disease mention with relation tags
  - DPNS: sequence of dependency nodes from chemical mention to disease mention
  - VBLS: sequence of verb lemmas from chemical mention to disease mention
  - WCDD: whether chemical mention and disease mention connect directly
  - WRCD: whether root and chemical mention connect directly
  - WRDD: whether root and disease mention connect directly

#### Feature extraction for inter-sentence level

#### 

Since two mentions occurred in different sentences, only lexical features could be used for inter-sentence level.

♦ Lexical features (LEX):
  - CBOW: bag-of-words of chemical mention
  - CPOS: part of speech of chemical mention
  - DBOW: bag-of-words of disease mention
  - DPOS: part of speech of disease mention
  - SDIST: sentence distance between chemical mention and disease mention
  - CFRQ: chemical frequency in document
  - DFRQ: disease frequency in document
  - WCO: whether the chemical is the only chemical entity in the document
  - WDO: whether the disease is the only disease entity in the document
  - SMBLOCK: whether the chemical and disease mentions occur in the same text block, e.g. ‘BACKGROUND’ section, ‘CONCLUSIONS’ section.

### Relation merging

After the relation extraction at mention level, we need to merge the results to acquire final relations between entities at document level. One assumption was that a pair of entities could have multiple pairs of mentions at intra-sentence level or inter-sentence level, and if at least one pair of these mentions explicitly supported the CID relationship, we would believe the two entities have the true CID relation.

### Post-processing

After relation merging, we employed the MeSH-controlled vocabulary again to resolve the redundancy caused by the hypernym relationship among the instances extracted from the same document. The main idea was similar to the discussion in Section ‘Hypernym filtering for training instances’ except that it was applied to the test instances rather than to the training instances.

## Results and discussion

In this section, we first present a brief introduction to the CDR corpus, and then we systematically evaluate the performance of our approach on the corpus.

### Dataset

The CDR corpus contained a total number of 1500 articles (only titles and abstracts) (20–22). The corpus was further split into three subsets: training, development and test sets with 500 articles for each. The training and development sets as well as 400 articles of the test set were randomly selected from the CTD-Pfizer corpus (4), which was generated from a collaborative biocuration process (23). The remaining 100 articles in the test set had not been curated previously by CTD, and were selected through a process similar to the previous CTD curation process in order to ensure the similar relation distribution with the training and development datasets.

**Figure 2.**
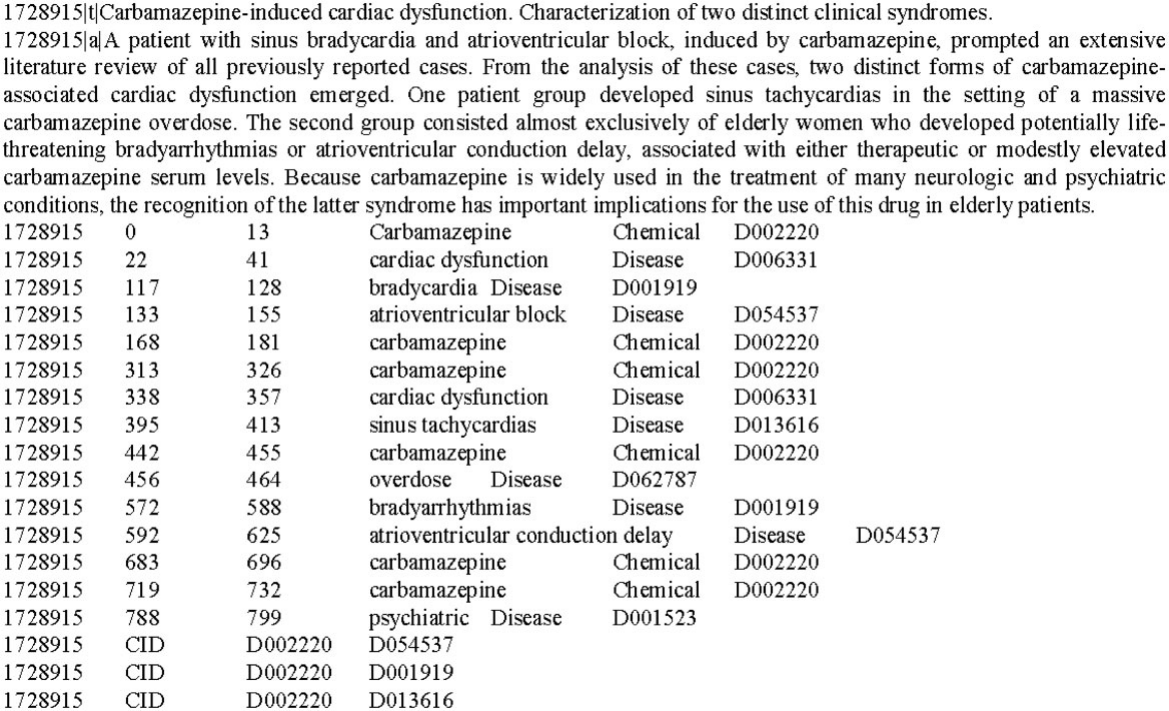
An annotation example of the CDR corpus.

Figure 2 shows the annotation format of the document (PMID: 1728915). The first row shows the title, and the second row shows the abstract. The ‘|t|’ and ‘|a|’ are separators used to distinguish title and abstract texts from entity and relation annotations. The rows below the abstract are entity mentions. Each mention is annotated with six attributes separated by the Tab keys, i.e. ‘*PMID < tab > START OFFSET < tab > END OFFSET < tab> MENTION TEXT < tab > MENTION TYPE (*i.e. *Chemical or Disease)<tab > IDENTIFIER*’. The ‘START OFFSET’ is the offset of the first character in the mention while the ‘END OFFSET’ is the offset of the last character. The CID relation is annotated at document level using concept identifiers to express the relationship. Each relation is annotated with four attributes separated by the Tab keys, i.e. ‘*PMID < tab > RELATION TYPE < tab > CHEMICAL IDENTIFIER < tab > DISEASE IDENTIFIER*’.

Table 1 shows the statistics on the numbers of articles and relations in the corpus. From the table, we can see that the three datasets have similar numbers of CID relations, which makes the corpus more balanced for training and evaluation.

**Table 1.** The CID relation statistics on the corpus

Task Datasets	No. of Articles	No. of CID Relations
Training	500	1038
Development	500	1012
Test	500	1066

### Preprocessing and evaluation metrics

As the first step, the tokenization of biomedical text is non-trivial and will significantly affect natural language processing pipelines (24). In this article, a simple tokenizer in (24) was adopted, which could break words into either a contiguous block of letters and/or digits or a single punctuation mark. For example, the string ‘*Lidocaine-induced*’ would be split into three tokens: ‘Lidocaine’, ‘-’, and ‘induced’. Although it led to larger number of tokens, the tokenization was considered as a standard process in natural language processing and made the dataset highly consistent with downstream components (24). After tokenization, the Stanford CoreNLP Tools (25) were used for sentence splitting, part-of-speech tagging and lemmatization.

In order to get dependency structures for sentences, we first employed the BLLIP reranking parser (26) (aka Charniak–Johnson parser) with *GENIA + PubMed* model (https://github.com/BLLIP/bllip-parser/) to obtain the constituent syntactic parsing trees, then we extracted the dependency structures from these constituent syntactic structures. In order to avoid entity mentions being split into multiple parts during the parsing process, we renamed the mentions in the form of ‘*DocumentID_startOffset_endOffset_mentionType*’. For instance, the sentence (*a*) in Section ‘Introduction’ would be processed as the following form before fed to the BLLIP parser:

After 2 individuals with Doc_10328196_309_318_Disease developed a Doc_10328196_331_354_Disease following treatment with oral Doc_10328196_385_394_Chemical lesional skin cells and activated peripheral blood cells were analyzed for induction of apoptosis.

For the classifier, the Mallet MaxEnt classifier (27) was adopted because of its universality and effectiveness for classification problems, and all the parameters remained as default.

Experiments were evaluated by commonly used metrics of the Precision (P), Recall (R) and harmonic *F*-score (F1). These metrics are calculated based on the numbers of true positives (TP), false positives (FP) and false negatives (FN) returned by the system:

(1) $$\text{Precision}=\frac{\text{TP}}{\text{TP}+\text{FP}}$$

(2) $$\text{Recall}=\frac{\text{TP}}{\text{TP}+\text{FN}}$$

(3) $$F\text{-score}=\frac{\text{2}\cdot \text{Precision}\cdot \text{Recall}}{\text{Precision}+\text{Recall}}$$

In addition, since there was no manual annotation available at intra- or inter-sentence level, it is hard to evaluate the extraction performance at intra- and inter-sentence levels, respectively. Therefore, we approached this problem in an approximate way based on the assumption in Section ‘Relation instance construction’. After the step of instance construction, a relation instance at mention level would be automatically labeled as true if the gold annotation was true between its chemical and disease entities, otherwise as false. This method was essentially similar to the distant supervision (28), which is usually used to automatically generate training examples from database records. The examples generated in this way usually contain noise. Nevertheless, it is a simple way to evaluate the extraction performance at mention level by taking these automatically generated labels as ground truth.

After merging the results at both intra- and inter-sentence levels, the evaluation for global performance was performed by comparing the system final output against the gold annotation at document level.

### Experimental results

Our experimental results are presented in the following order:

- Evaluation of the various linguistic features on the development dataset.
- Evaluation on the test dataset, and comparison with the related works.

#### Performance on the development dataset

#### 

Table 2 compares the performance of relation extraction at intra-sentence level and inter-sentence level, as well as the performance of the final results at document level on the development set using gold entity annotations. ‘LEX’, ‘DEP’ and ‘HF’ denote lexical features, dependency features and the hypernym filtering step mentioned in Section ‘Methods’. When comparing different levels of relations, the approach only using the lexical features is regarded as the baseline.

**Table 2.** Performance on the development dataset

Methods	Intra-sentence level			Inter-sentence level			Final CID Relation		
	*P*(%)	*R*(%)	*F*(%)	*P*(%)	*R*(%)	*F*(%)	*P*(%)	*R*(%)	*F*(%)
LEX (baseline)	**68.0**	59.2	63.3	46.0	33.1	38.5	58.1	52.7	55.3
DEP	67.7	54.2	60.2	–	–	–	58.7	51.2	54.7
HF+LEX	66.7	65.5	66.1	**46.2**	**39.0**	**42.3**	57.4	58.6	58.0
HF+DEP	65.5	61.8	63.6	–	–	–	56.6	57.4	57.0
HF+LEX+DEP	67.7	**67.6**	**67.7**	–	–	–	58.3	**60.1**	59.2
Post-Processing	–	–	–	–	–	–	**61.9**	59.0	**60.4**

Note: The best scores in each numerical column are in bold.

Note that DEP was unavailable for inter-sentence level, while HF and LEX could be applied to both levels. Post-processing was executed at document level based on the optimal feature combination after the relation merging step, i.e. ‘HF+LEX + DEP’. The table indicates that:

- Only using the lexical features, the final performance of *F*-score was able to reach as high as 55.3%, and the performance at intra-sentence level was much higher than that at inter-sentence level. This suggests that lexical features were simple yet more effective for intra-sentence level than for inter-sentence level. This is probably because the CID relations at inter-sentence level spanned several sentences and thus had much more complex structures that the traditional lexical features could not capture effectively.
- Though the performance by dependency features was slightly lower than that by lexical features, its *F*-score still reached as high as 60.2%. This is probably because of its capability to represent the direct syntactic relationships between different entity mentions in a sentence.
- On the basis of lexical or dependency features, hypernym filtering significantly improved the recall for both intra- and inter-sentence levels, leading to the *F*-scores of 66.1% and 42.3% for two levels, respectively. This indicates that filtering the more general negative instances from the training set caused more true relation instances to be recalled, justifying our hypothesis in the Section ‘Hypernym filtering for training instances’.
- Combining HF, LEX and DEP, our system achieved the best performance for relation extraction. After merging the relations from mention level to document level, the *F*-score reached as high as 59.2%.
- After post-processing, the *F*-score further reached as high as 60.4%. The minor decrease in the recall may be caused by the fact that there were some false annotations for the relations with more general entities.

To understand why the task is challenging, we have closely examined the errors and grouped the reasons as follows:

■ For intra-sentence level:
  - Lexical sparsity: Sentences that describe the CID relations using rarely occurring words may not be captured effectively. For instance, in the sentence ‘*Fatal* ***haemorrhagic myocarditis*** *secondary to* ***cyclophosphamide*** *therapy.*’ (PMID: 11271907), the key clue ‘… *secondary to* …’ occurs less frequently in the corpus.
  - The structure of sentence is complicated: If a sentence has a complicated structure, our method may not extract the CID relations correctly. For instance, in the sentence ‘*The epidemiologic findings are most consistent with the hypothesis that chronic* ***cocaine*** *use disrupts dopaminergic function and, when coupled with recent cocaine use, may precipitate agitation, delirium, aberrant thermoregulation, rhabdomyolysis, and* ***sudden death****.*’ (PMID: 8988571), though the relation between ‘***cocaine***’ (D003042) and ‘***sudden death***’ (D003645) is true, the token distance is too long and there are conjunction structures between mentions in the sentence.
  - True relations are neglected in annotation: A close-up analysis on the results shows that some of our false-positive predictions are actually true-positive. For instance, in the sentence ‘*This increase in aggressiveness was not secondary to* ***METH****-induced* ***hyperactivity****.*’(PMID: 16192988), the relation between ‘***METH***’ (D008694) and ‘***hyperactivity***’ (D006948) was extracted by our system. This relation is not annotated in the document; however, it is actually annotated in the documents of PMID: 15764424 and PMID: 10579464.
  - Inconsistent annotation: Correlated with the same entity, some relations are annotated while others are not. For instance, in the sentence ‘*One patient group developed sinus tachycardias in the setting of a massive* ***carbamazepine overdose****.*’(PMID: 1728915), the relation between ‘***carbamazepine***’ ****(D002220) and ‘***overdose***’ (D062787) is not annotated; however, in the sentence ‘*The possibility of choreoathetoid movements should be considered in patients presenting after* ***pemolineoverdose****.*’(PMID: 9022662), the relation between ‘***pemoline***’ (D010389) and ‘***overdose***’ (D062787) is annotated.
■ For inter-sentence level:
  - Discourse inference is needed: This is the most common error type at inter-sentence level. The inter-sentence level relations are expressed spanning multiple sentences, thus discourse inference including co-reference resolution is needed for the relation extraction. For instance, in two sentences ‘*Adverse events considered to be related to* ***levofloxacin*** *administration were reported by 29 patients (9%). The most common drug-related adverse events were diarrhea*, ***flatulence****, and nausea; most adverse events were mild to moderate in severity.*’ The relation between ‘***levofloxacin***’ (D064704) and ‘***flatulence***’ (D005414) is true, while the phrase of ‘*Adverse events*’ is the anchor bridging the two entities.
  - Inconsistent annotation: Correlated with the same entity, some relationships are annotated while others are not. This problem is similar to that at intra-sentence level.

#### Performance on the test dataset

#### 

During the BioCreative-V CID subtask, since the online evaluation only concerned the relation extraction results based on named entity recognition for participants, it is necessary to report our performance on the test dataset using the gold-standard entity annotations to exclude the influence from the named entity recognition step. Table 3 reports the performance of our approach on the test dataset using the gold entity annotations. The classification models were trained on the combination of both training and development datasets. The table shows that the performance scores were similar to that on the development dataset. However, the performance of inter-sentence level was much lower than that on the development set.

**Table 3.** Performance on the test dataset

Results	*P*(%)	*R*(%)	*F*(%)
Intra-sentence level	67.4	68.9	68.2
Inter-sentence level	51.4	29.8	37.7
Final CID Relation	62.0	55.1	58.3

#### Comparison with other systems

In Table 4, we compare our current results with the top two systems (29, 30), the official benchmarks and our official results (20, 31) when participating in the BioCreative-V online evaluation. Both of our current and online systems employed tmChem (10) and DNorm (11, 32), the state-of-the-art named entity recognizers for chemicals and diseases, respectively. In particular, the best official *F*-score of DNorm is 78.2%. Since there are two different models of tmChem whose best performances of *F*-scores are 86.6% and 87.4%, respectively, the second model was adopted in our system to recognize chemicals.

**Table 4.** Comparisons with the related works

Methods	RT	No. of TP	No. of FP	No. of FN	*P*(%)	*R*(%)	*F*(%)
Abstract level	–	815	4145	251	16.4	76.5	27.1
Sentence level	–	570	1672	496	25.4	53.5	34.5
Xu et al. (27)	8	623	496	443	55.7	58.4	57.0
Pons et al. (28)	16	574	544	492	51.3	53.8	52.6
Our online results	5	358	346	708	50.9	33.6	40.5
Approach in this article	13	439	355	627	55.3	41.2	47.2

In the table, RT stands for the response time of different systems, TP stands for the true-positive relations, FP stands for the false-positive relations and FN stands for the false-negative relations.

Xu et al. (29) employed various drug-side-effect resources to generate knowledge-based features as well as ngram word features for training a Support Vector Machine (SVM) classifier to extract CID relations. In particular, when training the classifier they took advantage of relation labels of chemical and disease pairs in the CTD (4) knowledge base, from which the CDR corpus was mainly generated.

Pons et al. (30) also used prior knowledge about chemicals and diseases to generate knowledge-based features with a fine tuned SVM classifier. They utilized the direct and indirect relation paths between chemicals and diseases in the knowledge base of BRAIN (33) when extracting CID relations.

During the online evaluation, our online system used the simplest lexical features without any dependency parsing or hypernym considerations, for the purpose of returning results in time to the evaluation server.

For the CID subtask, the BioCreative-V organizers implemented a co-occurrence-based baseline with two variants: abstract-level and sentence-level, where chemicals and diseases were automatically recognized by tmChem and DNorm, respectively.

From the table, we can observe that the co-occurrence method yielded a relatively high recall of 76.5%, but a drastically low precision of 16.4%. Since knowledge bases contain much manually refined information, they remarkably benefit the relation extraction task. Leveraging knowledge-based features, Pons et al. (30) achieved the *F*-score of 52.6%. It is worth noting that the knowledge in CTD (4) seems more powerful, and Xu et al. (29) achieved the highest *F*-score of 57.0%.

Compared with the official benchmarks, our online system significantly improved the precision, but with a lower recall. However, the approach presented in this article exhibited a promising improvement in precision as well as in recall, and the *F*-score finally reached as high as 47.2%. The response time of our current results was 13 s, much longer than our online system, due to the time-consuming syntactic parsing process. Compared with the top two systems, although our system did not achieve the comparable performance, it was more robust and did not rely on the domain-specific knowledge, and these advantages would make our system easier for generalization.

## Conclusion and future work

This article describes a supervised learning approach to automatically extract CID relations by using various linguistic features such as lexical and dependency information. In addition, the system leverages the MeSH-controlled vocabulary to help train the classifiers and address the problem of relation redundancy during the extraction process.

Our research exhibits promising results for relation extraction in the biomedical literature. Nevertheless, more work needs to be done to further improve the system performance. In the future, we plan to include richer information such as knowledge bases (Wikipedia, UMLS, SIDER, etc.), incorporate more data from publicly available databases and employ more powerful machine learning methods to achieve better results. The source code and the outputs of our experiments are available at https://github.com/JHnlp/BC5CIDTask.

## Funding

This research is supported by the National Natural Science Foundation of China [Grant No. K111817913, 61373096 and 90920004]. Funding for open access charge: National Natural Science Foundation of China Grant No. K111817913.

## References

[1] Dogan R.I. Murray G.C. Neveol A (2009) Understanding PubMed^®^ user search behavior through log analysis. Database, doi: 10.1093/database/bap018. 10.1093/database/bap018 PMC2797455 20157491

[2] Lu Z. (2011) PubMed and beyond: a survey of web tools for searching biomedical literature. Database, doi: 10.1093/database/baq036. 10.1093/database/baq036 PMC3025693 21245076

[3] Neveol A. Dogan R.I. Lu Z. (2011) Semi-automatic semantic annotation of PubMed queries: a study on quality, efficiency, satisfaction. J. Biomed. Info., 44, 310–318. 10.1016/j.jbi.2010.11.001 PMC3063330 21094696

[4] Davis A.P. Murphy C.G. Saraceni-Richards C.A (2009) Comparative Toxicogenomics Database: a knowledgebase and discovery tool for chemical-gene-disease networks. Nuc. Aci. Res., 37, D786–D792. 10.1093/nar/gkn580 PMC2686584 18782832

[5] Davis A.P. Grondin C.J. Lennon-Hopkins K. (2014) The Comparative Toxicogenomics Database’s 10th year anniversary: update 2015. Nuc. Aci. Res., 43, D914–D920. 10.1093/nar/gku935 PMC4384013 25326323

[6] Xu R. Wang Q. (2014) Automatic construction of a large-scale and accurate drug-side-effect association knowledge base from biomedical literature. J. Biomed. Info., 51, 191–199. 10.1016/j.jbi.2014.05.013 PMC4589180 24928448

[7] Kang N. Singh B. Bui C (2014) Knowledge-based extraction of adverse drug events from biomedical text. BMC Bioinfo., 15, 64. 10.1186/1471-2105-15-64 PMC3973995 24593054

[8] Krallinger M. Vazquez M. Leitner F (2011) The Protein-Protein Interaction tasks of BioCreative III: classification/ranking of articles and linking bio-ontology concepts to full text. BMC Bioinformatics, 12(Suppl 8), S3. 22151929 10.1186/1471-2105-12-S8-S3 PMC3269938

[9] Gurulingappa H. Mateen-Rajput A. Toldo L. (2012) Extraction of potential adverse drug events from medical case reports. J. Biomed. Info., 3, 15. 10.1186/2041-1480-3-15 PMC3599676 23256479

[10] Leaman R. Wei C.H. Lu Z. (2015) tmChem: a high performance approach for chemical named entity recognition and normalization. J. Cheminfo., 7, S3. 10.1186/1758-2946-7-S1-S3 PMC4331693 25810774

[11] Leaman R. Dogan R.I. Lu Z. (2013) DNorm: disease name normalization with pairwise learning to rank. Bioinfo., 29, 2909–2917 10.1093/bioinformatics/btt474 PMC3810844 23969135

[12] Xu R. Wang Q. (2013) Large-scale extraction of accurate drug-disease treatment pairs from biomedical literature for drug repurposing. BMC Bioinfo., 14, 181. 10.1186/1471-2105-14-181 PMC3702428 23742147

[13] Chen E.S. Hripcsak G. Xu H (2008) Automated acquisition of disease–drug knowledge from biomedical and clinical documents: an initial study. J. Am. Med. Info. Assoc., 15, 87–98. 10.1197/jamia.M2401 PMC2274872 17947625

[14] Wiegers T.C. Davis A.P. Mattingly C.J. (2014) Web services-based text-mining demonstrates broad impacts for interoperability and process simplification. Database, doi: 10.1093/database/bau050. 10.1093/database/bau050 PMC4207221 24919658

[15] Wiegers T.C. Davis A.P. Mattingly C.J. (2012) Collaborative biocuration-text-mining development task for document prioritization for curation. Database, doi: 10.1093/database/bas037. 10.1093/database/bas037 PMC3504477 23180769

[16] Wei C.H. Kao H.Y. Lu Z. (2013) PubTator: a Web-based text mining tool for assisting Biocuration. Nuc. Aci. Res., 41, W518–W522. 10.1093/nar/gkt441 PMC3692066 23703206

[17] Wei C.H. Harris B.R. Li D (2012) Accelerating literature curation with text-mining tools: a case study of using PubTator to curate genes in PubMed abstracts. Database, doi: 10.1093/database/bas041. 10.1093/database/bas041 PMC3500520 23160414

[18] Li J. Sun Y. Johnson R (2015) Annotating chemicals, diseases, and their interactions in biomedical literature. In: *Proceedings of the fifth BioCreative Challenge Evaluation Workshop*. BioCreative Organizing Committee. Sevilla, Spain, 173–182.

[19] Zhou G.D. Su J. Jie Z (2005) Exploring various knowledge in relation extraction. In: *Proceedings of the 43rd Annual Meeting of the Association for Computational Linguistics*. Association for Computational Linguistics. Michigan, USA, 427–434.

[20] Wei C.H. Peng Y.F. Leaman R (2015) Overview of the BioCreative V chemical disease relation (CDR) task. In: *Proceedings of the fifth BioCreative Challenge Evaluation Workshop*. BioCreative Organizing Committee. Sevilla, Spain, 154–166.

[21] Wei C.H. Peng Y.F. Leaman R (2016) Assessing the state of the art in biomedical relation extraction: Overview of the BioCreative V Chemical Disease Relation (CDR) Task. Database (manuscript in review). 10.1093/database/baw032 PMC4799720 26994911

[22] Li J. Sun Y. Johnson R (2016) BioCreative V CDR Task Corpus: a resource for chemical disease relation extraction. Database (manuscript in review). 10.1093/database/baw068 PMC4860626 27161011

[23] David A.P. Wiegers T.C. Roberts P.M (2013) A CTD-Pfizer collaboration: manual curation of 88,000 scientific articles text mined for drug-disease and drug-phenotype interactions. Database, doi: 10.1093/database/bat080. 10.1093/database/bat080 PMC3842776 24288140

[24] Leaman R. Gonzalez G. (2008) BANNER: An executable survey of advances in biomedical named entity recognition. Pac. Sym. Biocom., 13, 652–663. 18229723

[25] Manning C.D. Surdeanu M. Bauer J (2014) The Stanford CoreNLP Natural Language Processing Toolkit. In: *Proceedings of 52nd Annual Meeting of the Association for Computational Linguistics*. Association for Computational Linguistics. Baltimore, USA, 55–60.

[26] McClosky D Charniak E. (2008) Self-training for biomedical parsing. In: *Proceedings of 46th Annual Meeting of the Association for Computational Linguistics*. Association for Computational Linguistics. Columbus, USA, 101–104.

[27] McCallum A.K. (2002) MALLET: a machine learning for language toolkit. http://mallet.cs.umass.edu (accessed on 16.03.2016).

[28] Mintz M. Bills S. Snow R (2009) Distant supervision for relation extraction without labeled data. In: *Proceedings of the Joint Conference of the 47th Annual Meeting of the ACL and the 4th International Joint Conference on Natural Language Processing of the AFNLP*. Association for Computational Linguistics. Suntec, Singapore, 1003–1011.

[29] Xu J. Wu Y. Zhang Y (2015) UTH-CCB@BioCreative V CDR Task: Identifying Chemical-induced Disease Relations in Biomedical Text. In: *Proceedings of the fifth BioCreative Challenge Evaluation Workshop*. BioCreative Organizing Committee. Sevilla, Spain, 254–259.

[30] Pons E. Becker B.F. Akhondi S.A (2015) RELigator: chemical-disease relation extraction using prior knowledge and textual information. In: *Proceedings of the fifth BioCreative Challenge Evaluation Workshop*. BioCreative Organizing Committee. Sevilla, Spain, 247–253.

[31] Gu J.H. Qian L.H Zhou G.D. (2015) Chemical-induced disease relation extraction with lexical features. In: Proceedings of the fifth BioCreative Challenge Evaluation Workshop. BioCreative Organizing Committee. Sevilla, Spain, 220–225.

[32] Leaman R. Lu Z. (2014) Automated disease normalization with low rank approximations. In: *Proceedings of BioNLP* Association for Computational Linguistics. Baltimore, USA, 593, 24.

[33] Bio-IT World. Big BRAIN: finding connections in the literature flood with Euretos BRAIN. http://www.bio-itworld.com/2014/7/1/big-brain-finding-gems-literature-flood-euretos-brain.html (accessed on 16.03.2016).

